# Emerging phleboviruses^☆^

**DOI:** 10.1016/j.coviro.2014.01.011

**Published:** 2014-04

**Authors:** Richard M Elliott, Benjamin Brennan

**Affiliations:** MRC-University of Glasgow Centre for Virus Research, 464 Bearsden Road, Glasgow G61 1QH, Scotland, UK

## Abstract

The *Bunyavidae* family is the largest grouping of RNA viruses and arguably the most diverse. Bunyaviruses have a truly global distribution and can infect vertebrates, invertebrates and plants. The majority of bunyaviruses are vectored by arthropods and thus have the remarkable capability to replicate in hosts of disparate phylogeny. The family has provided many examples of emerging viruses including Sin Nombre and related viruses responsible for hantavirus cardiopulmonary syndrome in the Americas, first identified in 1993, and Schmallenberg virus which emerged in Europe in 2011, causing foetal malformations in ruminants. In addition, some well-known bunyaviruses like Rift Valley fever and Crimean-Congo haemorrhagic fever viruses continue to emerge in new geographical locations. In this short review we focus on newly identified viruses associated with severe haemorrhagic disease in humans in China and the US.

## Highlights

• New phleboviruses (family Bunyaviridae) have emerged in China and the US.
• Tick borne phleboviruses do not encode an NSm protein.
• Tick borne phleboviruses show differential pathogenicity in humans.

**Current Opinion in Virology** 2014, **5**:50–57

This review comes from a themed issue on **Emerging viruses**

Edited by **Christopher F Basler** and **Patrick CY Woo**

For a complete overview see the Issue and the Editorial

Available online 28th February 2014

1879-6257/$ – see front matter, © 2014 The Authors. Published by Elsevier B.V. All rights reserved.

**http://dx.doi.org/10.1016/j.coviro.2014.01.011**

## Introduction

Bunyaviruses are characterised by the possession of a tri-segmented negative-sense RNA genome. Viruses are enveloped, replicate in the cytoplasm and mature by budding at the Golgi. Virions are about 100 nm in diameter and are composed of just four proteins: two glycoproteins termed Gn and Gc that are embedded in the Golgi-derived viral membrane and two internal proteins, the nucleocapsid (N) protein that encapsidates each of the three genome segments in the form of ribonucleoprotein complexes, and the L protein (RNA dependent RNA polymerase). The largest RNA segment, L, encodes the L protein, the medium segment, M, the glycoproteins, and the smallest segment, S, the N protein. In addition some viruses encode up to three nonstructural proteins [1,2]. The more than 350 named bunyaviruses are subdivided into five genera (*Orthobunyavirus, Hantavirus, Nairovirus, Phlebovirus* and *Tospovirus*) on serological, morphological and biochemical features [3]. There are differences in the patterns of sizes of the viral RNAs and structural proteins between the different genera, and the expression strategy of non-structural proteins also differs between different genera. In addition, there are consensus terminal sequences of the genome segments that are conserved on a genus specific manner; for phleboviruses these are 5′-ACACAAAG… and …CUUUGUGU-3′.

## The Phlebovirus genus

**Figure 1:**
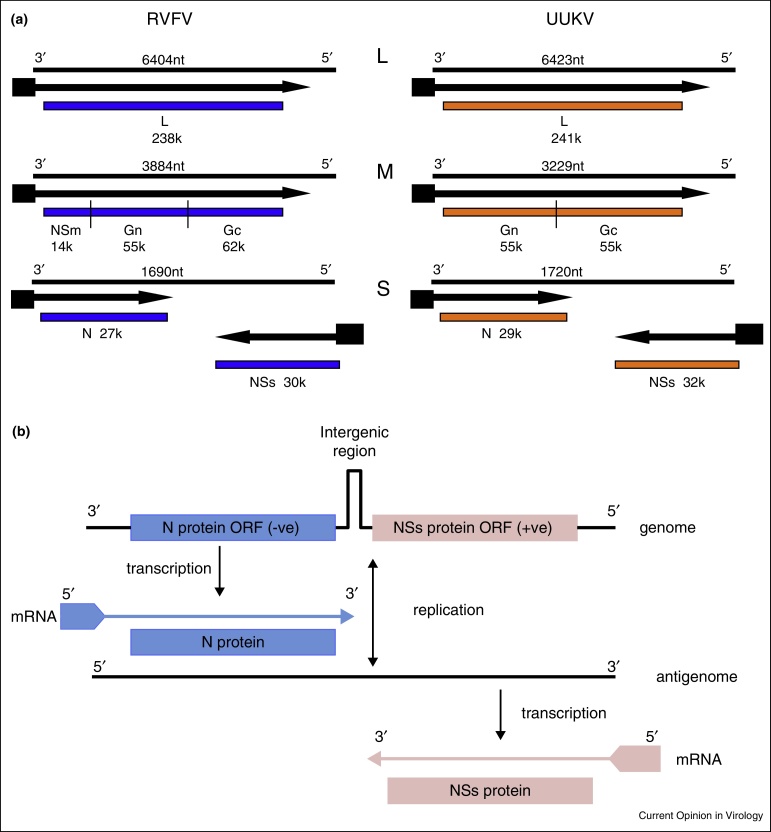
The phlebovirus genome. **(a)** Comparison of the coding strategy of the sandfly fever group (Rift Valley fever, RVFV) and Uukuniemi group (UUKV) genomes. RNAs are represented by thin lines (the length in nucleotides is given above each segment) and the mRNAs are shown as arrows (■ indicates host-derived sequences at 5′ end). Gene products, with their apparent *M*_r_, are represented by coloured boxes. **(b)** Transcription and replication scheme of ambisense-sense phlebovirus S genome segment. The genome RNA encodes the N protein in the negative-sense and the NSs protein in positive-sense orientation, separated by an intergenic region that has the potential to form a hairpin structure. The proteins are translated from specific sub-genomic mRNAs, with the mRNA encoding NSs transcribed from the antigenome RNA.

In the latest report of the International Committee on Taxonomy of Viruses (ICTV) [3] the *Phlebovirus* genus contains 70 viruses, which comprise 9 species and 33 tentative species, and can be divided into two groups, the sandfly fever virus group and the Uukuniemi-like virus group (Table 1). Before 1991, the Uukuniemi-like viruses were in a separate genus (*Uukuvirus*), but the similar molecular biological characteristics along with some weak serological cross reactivity shared with viruses in the *Phlebovirus* genus resulted in their reclassification [4]. There are some important differences between these viruses, notably that the Uukuniemi-like viruses do not encode a nonstructural (NSm) protein at the N-terminus of the glycoprotein precursor coding region (Figure 1a), and that the Uukuniemi-like viruses are all transmitted by ticks whereas the sandfly fever group are transmitted by dipterans (phlebotomines and mosquitoes) [5].

A characteristic of all phleboviruses is the ambisense coding strategy of the S genome segment: the N protein is encoded in the negative-sense orientation on the S segment while the NSs protein is encoded in the positive-sense. However, both proteins are translated from separate subgenomic mRNAs that are transcribed from the genomic or antigenomic RNA as shown in Figure 1b [6].

The best-known phlebovirus is Rift Valley fever virus, a serious pathogen of ruminants but also capable of causing disease, sometimes fatal, in man [7,8]. Other human pathogens include sandfly fever Naples, sandfly fever Sicilian and Toscana viruses that cause febrile illness and occasionally encephalitis, and are found in countries of the Mediterranean basin. On the other hand, the tick-transmitted Uukuniemi and Uukuniemi-like viruses are non pathogenic for humans [5]. Recently, new tick-transmitted phleboviruses have been described, called severe fever with thrombocytopenia syndrome virus and Heartland virus, that can cause serious disease in humans.

## Severe fever with thrombocytopenia syndrome virus

Between 2007 and 2010 cases of an unknown infectious disease were reported in Henan and Hubei Provinces, China, with patients presenting gastrointestinal symptoms, chills, joint pain, myalgia, thrombocytopenia, leukocytopenia and some haemorrhagic manifestations, resulting in a case fatality rate of 12–30% [9^••^]. The disease was originally suspected to be anaplasmosis, but some clinical symptoms were inconsistent with this diagnosis. Subsequently, studies by different groups in China involving virus isolation in cell culture, genome amplification and sequencing, and metagenomic analysis of patient material revealed the presence of a novel bunyavirus that was most closely related to phleboviruses. The virus has been variously called DaBie Mountain virus [9^••^,10], Henan fever virus [11], Huaiyangshan virus [12] and severe fever with thrombocytopenia syndrome virus (SFTSV) [9^••^]. SFTSV is the name preferred by the Chinese CDC and will used here, although the ICTV has yet to formally approve a name for this new phlebovirus.

**Figure 2:**
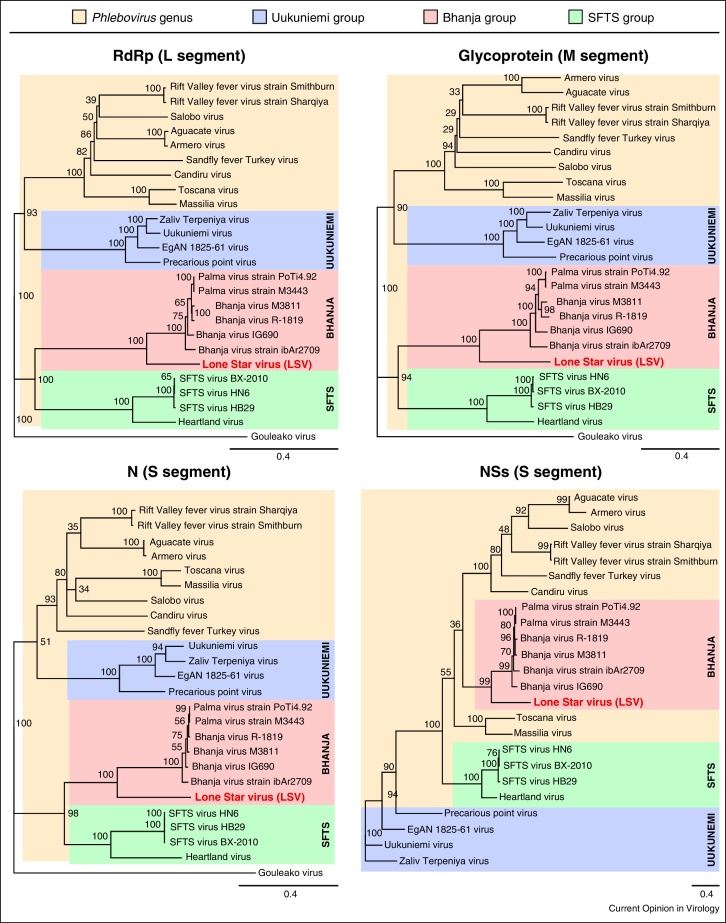
Phylogeny of representative phleovirus proteins. The analogous protein from Gouleako virus, a mosquito-associated phlebovirus-like virus, was used as the outgroup in each case. Taken from Swei *et al.* [38^•^].

Comparison of SFTSV viral proteins showed 33%, 30–36%, 30–41% and 11–13% similarity to either RVFV or UUKV RNA dependent RNA polymerase, glycoprotein precursor, N or NSs proteins respectively [9^••^]. Notably the glycoprotein precursor lacked a putative NSm protein at its N-terminus. Phylogenetic anlaysis of SFTSV sequences indicated that it represents a new clade within the *Phlebovirus* genus, roughly equidistant from the already established sandfly fever and Uukuniemi groups [9^••^] (Figure 2).

Both SFTSV and viral RNA have been isolated from *Haemaphysalis longicornis* ticks, and viral RNA has been detected in *Rhipicephalus microplus* ticks gathered from domestic animals in China [9^••^,13]. Detection of SFTSV RNA was highest in *H. longicornis*, a species that has a widespread geographical distribution outside of China including Korea, Japan, Australia, New Zealand and Pacific Islands [14]. The subsequent reports of SFTSV positive ticks and confirmed cases of disease in South Korea [15^•^] and Japan [16^•^] indicate that SFTSV and SFTSV-like viruses may have a broad distribution and further surveillance is warranted.

Many SFTSV genome sequences have been determined, and a deviation from the consensus terminal nucleotide sequence was observed at the 5′ termini of the L and M segments (Table 2). A comprehensive analysis of available sequences suggested that they could be separated into three major lineages (I to III), with two sublineages in lineage I [11]. Significantly, the M segment of one of the sublineages showed evidence for recombination, with two break points, between the M segments of lineage I and lineage III [17^•^]. This resulted in lineage I having acquired almost the complete coding sequence for Gn from lineage III. Homologous recombination is regarded as a rare event among negative-sense RNA viruses [18] though has previously been reported in the bunyavirus family, specifically hantaviruses [19]. Thus, in addition to the more usual evolutionary mechanisms of genetic drift and genome segment reassortment [20], recombination could also play a role in the evolution of SFTSV [11].

Research efforts are now focusing on understanding the molecular biology of the virus lifecycle and its pathogenesis in animal hosts. Qu *et al.* [21] found SFTSV able to infect a human monocytic cell line THP-1 in the absence of any discernable cytopathic effect (CPE) evidenced by a lack of caspase-3 cleavage, in contrast to the extensive CPE associated with RVFV infection [22]. Infection resulted in an up-regulation of interferon induction through IRF and NF-κB activation, though some regulatory molecules such as TRAF3, TRAF6 or MAVS were down-regulated or unchanged. It was further shown through the use of reporter assays that the NSs protein of SFTSV was responsible for this effect, and able to inhibit the induction of IFN. The NSs proteins of other phleboviruses as well as of orthbunyaviruses have also been shown to act as IFN antagonists [23]. An interaction of SFTSV NSs with Tank-binding kinase 1 (TBK-1) was described, suggesting a potential mechanism for the deregulation of the interferon response during infection as TBK-1, a homolog of IKKɛ, activates both NF-κB and IRF-3. The authors also reported a novel role for the SFTSV nucleocapsid protein (N) in the suppression of the IFN-β promoter [21], a function not previously reported for the N proteins of other members of the *Bunyaviridae*.

The crystal structure of SFTSV N protein has been solved by two groups [24^•^,25^•^]. Despite having a distinct amino acid sequence to that of RVFV, the SFTSV nucleocapsid protein also oligomerised to form a hexameric ring [26,27] to accomplish encapsidation of the genomic RNAs. Three amino acids (R64, K67 and K74) were identified to be crucial for the ability of the protein to bind RNA *in vitro* [24^•^], while mutations at residues A8, F11, A25, and L28 were shown to significantly hinder the ability of the protein to form oligomeric conformations [25^•^]. The interaction of the N-terminal arm of one protein with a neighbouring N protomer is predicted to be the mechanism by which the hexameric oligomerisation of the SFTSV nucleocapsid occurs. Indeed, deletion of the N-terminal 34 amino acids of SFSTV N disrupts the functional interactions of the N-terminal arm, and resulted in only monomeric protein being detected [25^•^]. Suramin was reported to inhibit SFTSV-N RNA binding by locating in the RNA binding cavity on N, and also had functionality against other members of the *Phlebovirus* genus, but not viruses in other *Bunyaviridae* genera. Suramin inhibited SFTSV replication in infected Vero E6 cells. [24^•^].

Using rhabdovirus-based vectors for expression of SFTSV Gn and Gc proteins, Hofmann *et al.* [28] demonstrated that SFTSV entered a wide range of cell lines, including human macrophage and dendritic cells, in a pH-dependent manner. Infection by the SFTSV pseudotypes could be abolished using sera from convalescent SFTS patients. Similar to RVFV [29], SFTSV utilizes the C-type lectin DC-SIGN as a receptor for entry into the cells [28].

These insights have been valuable to the understanding of SFTSV biology at the cell and molecular level. On the organismal level, several animal models of infection have been described to begin to understand the underlying pathologies associated with SFTSV infection such as thrombocytopenia and leukocytopenia. Wistar rats, Kunming mice, BalB/C mice, C57/BL mice, C57/BL6 mice and hamsters have been explored [30,31]. Newborn animals were highly susceptible to SFTSV infection, with the exception of newborn hamsters (no deaths resulted from either an intracerebral or intraperitoneal inoculation), while no reduction of white blood cells or platelets could be detected during infection of BalB/C mice or hamsters [30,31]. The C57/BL6 mouse model shows particular promise as SFTSV infection in these animals mimics the major clinical manifestations observed in infected patients and identified the major cause of thrombocytopenia to be an enhanced clearance of virus-infected platelets promoted by splenic macrophages. The authors suggest that SFTSV could bind to platelets and cause their recognition and phagocytosis by macrophages in the red pulp of the spleen [30].

## Heartland virus

Heartland virus was isolated from two patients in Missouri, USA [32^••^] who experienced fever, fatigue, anorexia, diarrhoea and thrombocytopenia. Both patients reported tick-bites prior to disease, and both recovered after extended hospitalisation. Deep-sequencing of total RNA from tissue culture cells inoculated with the patients’ blood revealed a novel phlebovirus that clustered phylogenetically with SFTSV (Figure 2). However HRTV is quite distinct from SFTSV, showing 27% and 38% difference in the RdRp and N protein sequences respectively. The two HRTV isolates showed 95-99% nucleotide identity to each other, depending on the segment, indicating that the patients were infected independently [32^••^]. The HRTV S and M segments showed a G residue rather than A at position 6 in the 5′ terminus (Table 2). HRTV RNA was subsequently detected in 10 pools of nymphs of *Amblyomma americanum* ticks (nine of which were found on the property of one of the patients) and virus has been isolated from 8 of the pools, implicating *A. americanum* as the likely primary vector for transmission of the virus within the USA [33].

## Bhanja virus

Bhanja virus (BHAV) is not a newly emerged virus but recent molecular characterisation of this and related viruses is pertinent to the above discussion of SFTSV and HRTV. The Bhanja virus antigenic complex (Bhanja, Forecariah, Kismayo and Palma viruses) comprises tick-borne viruses that were assigned to the family *Bunyaviridae* but were not further classified into a genus. BHAV was isolated in India in 1954 from a tick on a paralysed goat, and causes fever and signs of central nervous system involvement in young ruminants but not in adult animals. A few cases of febrile illness in humans have also been described. BHAV has been reported in southern and central Asia, Africa, and southern Europe (Italy, Croatia, Bulgaria, Slovakia, and Romania) [34]. Two recent papers report nucleotide sequence determination of Bhanja group viruses [35^•^,36^•^] and show that they are related to SFTSV and HRTV (Figure 2). Variations from the consensus terminal sequences were noted at the 5′ terminus of the L segment and the 3′ end of the S segment (Table 2). The Bhanja group viruses can be divided into African and Eurasian lineages possibly due to association with different tick vectors: the Eurasian viruses have been isolated from *Haemaphysalis* ticks, like SFTSV, whereas the African strains have been isolated from a wider range of tick species.

## Lone Star virus

Lone Star virus was originally isolated from *A. americanum* (the lone star tick) in Kentucky in 1967 [37], and like BHAV was an unclassified member of the *Bunyaviridae.* The sequence of the viral genome has recently been determined by deep sequencing and shown to be in the same clade as BHAV [38^•^] (Figure 2). The S segment 5′ terminus has a G residue at position 6 rather than A as found in the phlebovirus consensus sequence (Table 2). LSV can infect human (HeLa) and monkey (Vero) cells in culture but there is no evidence for human infection.

## Conclusions

The number of known phleboviruses has increased markedly in recent years with the emergence of SFTSV and HRTV, and the genetic characterisation of the previously unassigned BHAV, LSV and related viruses. These viruses form clades (Figure 2) that are related to the Uukuniemi-like viruses, and all share the properties of not encoding an NSm protein and being transmitted by ticks. For Rift Valley fever virus, the NSm protein is important for infectivity in *Aedes aegypti* mosquitoes [39] as a deletion mutant was barely able to establish infection. In addition, although not essential for growth in mammalian cells or animals [40–42] NSm regulates the p38 mitogen-activated protein kinase stress response [43] and has an antiapoptotic role [22], mediated by its interaction with the outer mitochondrial membrane [44]. Whether the Uukuniemi-like viruses inhibit apoptosis, and if so, which viral proteins perform this task, awaits further investigation.

It is likely that more relatives of these tick-borne phleboviruses will be discovered in the future, aided by advances in metagenomic analysis and perhaps heightened surveillance, as has been implemented in China. Of great importance is to understand the molecular basis for the different pathogenicity of these viruses in man, a task that will keep bunyavirologists occupied for years to come.

## References and recommended reading

Papers of particular interest, published within the period of review, have been highlighted as:

• of special interest
•• of outstanding interest

## Figures and Tables

**Table 1:** Species in the *Phlebovirus* genus according to current classification [3]

Species	Notable virus	Geographic distribution	Principal vector	Disease
*Bujaru virus*	Bujaru virus	South America	N.D.	

*Candiru virus*	Alenquer virus	South America	N.D.	Human
	Candiru virus	South America	N.D.	Human

*Chilibre virus*	Chilibre virus	North America	Phlebotomines	

*Frijoles virus*	Frijoles virus	North America	Phlebotomines	

*Punta Toro virus*	Punta Toro virus	North America, South America	Phlebotomines	Human

*Rift Valley fever virus*	Rift Valley fever virus	Africa	Mosquitoes	Human, cattle

*Salehabad virus*	Salehabad virus	Asia	Phlebotomines	

*Sandfly fever Naples virus*	Sandfly fever Naples virus	Europe, Africa, Asia	Phlebotomines	Human
	Sandfly fever Sicilian virus	Europe	Phlebotomines	Human
	Toscana virus	Europe	Phlebotomines	Human

*Uukuniemi virus*	Uukuniemi virus	Europe	Ticks	

**Table 2:** Nucleotide sequences of the 5′ and 3′ termini of phlebovirus antigenomic sense RNA segments. Bases deviating from the consensus are highlighted

Virus	Accession no.	Segment	5′ UTR	3′ UTR
RVFV	DQ375404	L	ACACAAAG…	…CUUUGUGU
	DQ380208	M	ACACAAAG…	…CUUUGUGU
	DQ380154^a^	S	ACACAAAG…	…CUUUGUGU

UUKV	D10759	L	ACACAAAG…	…CUUUGUGU
	M17417	M	ACACAAAG…	…CUUUGUGU
	M33551	S	ACACAAAG…	…CUUUGUGU

SFTSV	HM745930	L	ACACAGAG…	…CUUUGUGU
	HM745931	M	ACACAGAG…	…CUUUGUGU
	HM745932	S	ACACAAAG…	…CUUUGUGU

HRTV	JX005847	L	ACACAAAG…	…CUUUGUGU
	JX005845	M	ACACAGAG…	…CUUUGUGU
	JX005843^a^	S	ACACAGAG…	…CUUUGUGU

BHAV	JX961619	L	ACACAGAG…	…CUUUGUGU
	JX961620	M	ACACAAAG…	…CUUUGUGU
	JX961621	S	ACACAAAG…	…CUCUGUGU

LSV	KC589005	L	ACACAAAG…	…CUUUGUGU
	KC589006	M	ACACAAAG…	…CUUUGUGU
	KC589007	S	ACACAAAG…	…CUCUGUGU

a Sequence in database presented as genomic sense RNA.
